# Why the SAFE—*S* Strategy for Trachoma? Are *Musca sorbens* or *Scatophaga stercoraria* Really the Culprit?—A Brief Historical Review from an Italian Point of View

**DOI:** 10.3390/pathogens12121419

**Published:** 2023-12-04

**Authors:** Carla Enrica Gallenga, Martina Maritati, Marco Del Boccio, Rossella D’Aloisio, Pio Conti, Marco Mura, Carlo Contini, Pier Enrico Gallenga

**Affiliations:** 1 Department of Medical Science, University of Ferrara, Eye Clinic University-Hospital, 44124 Ferrara, Italy; 2 Department of Medical Science Infectious Diseases and Dermatology Section, University of Ferrara, 44124 Ferrara, Italy; mrtmtn@unife.it (M.M.); carlo.contini@unife.it (C.C.); 3 Centro Medico Santa Lucia, 66054 Vasto, Italy; gilbertodelboccio2019@gmail.com; 4 Department of Medical, Oral, and Biotechnological Sciences, G. d’Annunzio University Chieti-Pescara, 66100 Chieti, Italy; oftalmologia@unich.it; 5 Molecular Immunopharmacology and Drug Discovery Laboratory, Tufts University Medical School, Boston, MA 02111, USA; pioconti@yahoo.it; 6 Department of Traslational Medicine and for Romagna, Section Ophthalmology, University of Ferrara, 44124 Ferrara, Italy; marco.mura@unife.it; 7 Stenella cno Ophthalmology Laboratory, 65100 Pescara, Italy; studiogallenga@libero.it; 8 Bioethical Committee of San Marino Republic, 47893 Borgo Maggiore, San Marino

**Keywords:** trachoma, chlamydia, WHO SAFE strategy, RT-PCR, conjunctivitis, sexually transmitted diseases, neglected transmitted diseases, *Musca sorbens*, vaccines

## Abstract

The biological history of *Chlamydia trachomatis* is intertwined with the evolution of the man. Infecting Elemental Bodies (EBs), having penetrated mucosal epithelial cells, wrap themselves in a cloak (ĸλαμις) of glycogen that ensures their obligatory intracellular survival and protects this differentiation into Reticulate Bodies (RBs) that feed on cellular ATP. Multiple chemokines and cytokines are involved under the direction of IL-6 in the florid phase and IL-17A in the scar phase. The WHO has successfully identified the SAFE strategy against trachoma (Surgery, Antibiotics, Facial cleansing, Environment) as the blueprint to eliminate the disease by 2020. Recently, interest has been increasingly focused on changing sexual attitudes in different areas of the world, leaving *Musca sorbens*, *Scatophaga stercoraria*, and stepsisters fairly blameless, but extolling the role of *Chlamydia trachomatis* in apparently “sterile” chronic prostatitis or conjunctivitis or, less frequently, in oropharyngitis and proctitis. The addition of an *S* (SAFE-*S*) standing for “sexual behavior” was then proposed to also attract the interest and attention not only of Ophthalmologists and Obstetricians/Gynecologists, Urologists/Andrologists, and the School Authorities for information on the prevention of sexually transmitted diseases, but also of Social Physicians and Pediatricians. This means that sexually transmitted infections should be screened in asymptomatic patients with risky sexual behavior or sexual contact with people diagnosed with a transmitted infection.

## 1. History and Italian Background

**Figure 1:**
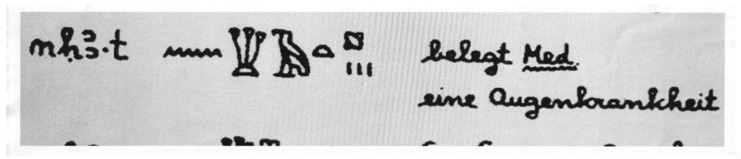
Eber papyrus, approximately 1550 BC, Egypt, New Kingdom, XVIII dynasty. Medical papyrus on diseases and herbal knowledge, written in hieratic as a *summa* of older texts. Line 350. “*nekha.t*” = this can be interpreted as ‘trachome’, conjunctivitic granulosis; *ripple of water* = “n” as a phoneme; *clumps of papyrus* = “kh” as a phoneme, sounds like “ch” in German; *vulture bird* = “a” as a phoneme (it is generally transliterated with sign “3” to indicate an open “A”); *bread loaf* = “t” as a phoneme; *pustule* is a determinative for blister, wound or phlogosis; *three vertical lines* = indicate a plural form (maybe a number of blisters or tearing). The word “nekha.t” is translated as “an eye disease” [belegt Med. eineAugenkrankheit] in Woerterbuch [the Dictionary]. (Cristiano Daglio. Le Malattie. S. Malgora: Ur Sunu, pp 65–81. Saviolo ed. Vercelli, Italy, 2008. Courtesy Dr. F. Buttiglione PhD and Serena Salmé).

There are mentions of trachoma, known since antiquity, in Hammurabi’s Code of the 18th century B.C., also carved into the diorite of the 12th century B.C. in the Sousa stele, now in the Louvre, and in the 16th century B.C. Eber’s Papyrus, now in Leipzig (Figure 1).

Knowledge of the School of Alexandria is reported in the *De re medica* by Celsus, who was active in Rome in the first century AD. In the *Epitome* book III *De Trachomate* by Paulus Aegineta, in the seventh century A.D., the florid granular aspect of the conjunctiva is described resembling a fig and defined as “sycosis”, while “tylosis” is the chronic form (for treatment: hematite, wine, saffron, Viergespann), but it remains generally confused in the set of conjunctivitis-secreting pathologies [1]. Francis of Assisi suffered severely on his return from Egypt, where he met Sultan Malik al Kamil in the summer truce at the siege of Damietta in 1219, during the Fifth Crusade. Infected and back in Italy, he was treated with eyelid burns with red-hot iron, not complaining but praising God for the sufferings: he died at 44, almost blind.

Mackenzie [2] in 1854 wondered if the disease “*est susceptible de se propager par infection, c’est à dire si les miasmes qui s’echappent des yeux de ceux qui en son affecés peuvent, en se repandant dans l’atmosphère, provoquer le développement de la meme affection dans d’autres yeux*”, while citing spreads in communities, barracks, schools and families and the possible transmission with infected towels. 

Patients were treated both as outpatient or inpatient in general surgeries or the medical hospital, together with syphilodermopathic patients, as in the Ophthalmic Hospital of Turin, Italy, founded by senator Prof. Casimiro Sperino, transforming in 1835, on behalf of Carlo Alberto King of Sardinia [3], the previous hospitals (the so called ‘lazzaretto’: pesthouse) laid for the cholera epidemic. 

There, of the 12,114 patients hospitalized in the decade 1873–1883 in the Ophthalmology Clinic of the R. University of Turin headed by Prof. Carlo Reymond, Dr. Camillo Gallenga published 1.392 cases of corneal leucoma including granular conjunctivitis, phlyctenular and pustular affections of the conjunctiva, ulcers and corneal abscesses “*of which often represent the final outcome*” [4], treating with Snellen’s procedure the entropion with trichiasis in severe forms of granular conjunctivitis [5]. 

Trachoma has been classified with etiological/microbiological identity in Ophthalmology treatises since the second World War, when the basophilic bodies of Halberstaedter (German dermatologist and radiologist) and von Prowazek (Austrian biologist/zoologist), identified in 1907 in Jakarta during a scientific expedition to study syphilis transmission [6], were definitively attributed to *Chlamydia trachomatis* (C*t*). Halberstaedter and von Prowazek, in an expedition to Java, also infected orangutans with conjunctival scrapings from trachoma patients and such agents in conjunctival smears. They called them “chlamydozoa”. 

Just two years later, in 1909, an early confirmation was published by Camillo Gallenga, actually Professor of Ophthalmology in the University of Parma [7], replicated the next year 1910 for the German literature in *Klinische Monatsblatter Augenheilkunde* [8], contrasting opposite skeptical opinions largely represented by Scientists and Ophthalmologists not only in Europe. 

Axenfeld [9], in 1914, still attributed the spread of trachoma in Europe to Napoleon’s Egyptian campaign, linked to granulomatous conjunctivitis, whose diagnosis became certain when it was confirmed by “*corpuscules trachomateux de Prowazek*” stained with Giemsa, accordingly with the 1907 first description of the etiology, supported by the Italian literature [7,8]. 

The May–Grunwald Giemsa solution stains chromatin in red-purple (Romanowsky effect), nucleoli in deep blue and the cytoplasm of epithelial cells in light blue. The smallest inclusions, even two or four in number, are generally associated in small aggregates with an intensely basophilic matrix and with a homogeneous surface element that differs from the cytoplasm and colors in intense blue, like a cap. Although differentiated, these Reticulated Bodies (RBs) tend to adhere to the nucleus. The initial inclusions are replaced by Elementary Bodies (EBs), which color red-purple and remain well separated from each other [10]. The amorphous masses of the blue-colored inclusions envelop like a cloak (κλαμίς: the ancient Greek term for the short cloak worn by Greek military men, draped and secured with a brooch on the right shoulder) the purple-red granules of chromatin ‘carriers of the virus’. The Chlamydiae were named this way because the intracytoplasmic inclusions formed by this agent inside host cells cluster around (are ‘draped’ around) the nucleus of the cell [11]. Still, in 1925, Amilcare Bietti discusses the distinction or unity of trachoma and ophthalmo-blennorrhea by inclusion bodies in children [12]. He reports—disputing the hypothesis—that Lindner, on the contrary, was convinced that the two affections were identical, although the latter was unable to infect the genitals of a female baboon with human ocular trachomatous material. Riccardo Gallenga, while operating the plasmoma of the superior bulbar conjunctiva of an eighteen-year-old boy, trachomatous from infancy and now in the scarring phase (Trachoma register int. N° 113 18, 1931, Ophthalmology Clinic R. University of Turin, head Prof. Luigi Guglianetti) in the accurate description of the histological examination, reports “*in the remarkably thickened epithelium... the presence of polynuclear leukocytes, lymphocytes and plasmacells*”, but “*I have not observed either epithelial cells or trachomatous granules*” [13] and describes, in the plasmome, the presence of “*rare Russel bodies colored in pink with eosin and in yellow with van Gieson*”: the reference documents the absence of Chlamydia in the bulbar conjunctiva of trachoma in the MacCallan cicatricial stage T4, and the precise knowledge of the results by Halberstäedter and von Prowazeck. 

The public health response to the spread of trachoma throughout the Mediterranean basin in the 19th and first half of the 20th century involved anti-trachoma clinics and the establishment of dispensaries in all major cities and hospitals. In all of them, there was intense activity and attendance. So much so that in the years 1936–1942, before and during the Second World War (but before the RAF and USAF heavily bombed the city, port and hospital of Cagliari, Sardinia), the flow of patients to the anti-trachoma department of the Cagliari University Eye Clinic headed by Professor Riccardo Gallenga was so intense that it had to be regulated every morning by the traffic police. There, in 1938, by suturing the conjunctival biopsy from surgery for entropion by a T4 patient to the mucosa of his own conjunctival fornix, Riccardo Gallenga also demonstrated that cicatricial trachoma is no longer contagious (Lessons, The Ophthalmology Course, 5th year Medical School University of Turin, 1966; and OMC&O Turin conference, University Eye clinic, 1967) [14], accordingly with his previous results as Assistant Professor in Turin Eye Clinic [13]. 

Meanwhile, still in Sardinia island, Prof. Andrea Contini, Director of the Experimental Zooprophylactic Institute of Sardinia and then full Professor of Microbiology and Infectious Diseases at the Faculty of Veterinary Medicine of the University of Sassari, made an important contribution. Scientifically trained at the Pasteur Institute in Paris under the direction of Prof. Lepine, a world-famous virologist (he developed and introduced his polio vaccine—‘Lepine vaccine’—in the early 1960s, which is still used in France today), Contini was able to isolate C*t* from conjunctival scrapings of children with florid trachomatosis. Starting from the application of simple light microscopy and using the chorioallantoic membrane of embryonated chicken eggs, he highlighted, after appropriate and specific staining (Macchiavello staining) the biological cycle of the pathogen, from penetration into the cell with its multiplicative phase, to the exit of the EB, ready to parasite other cells. He then succeeded in re-isolating the pathogen according to Koch’s postulates as incontrovertible proof of the truthfulness of the experiment and of the isolated etiological agent. The results were published in a prestigious journal [15] and in a book [16]. 

**Figure 2:**
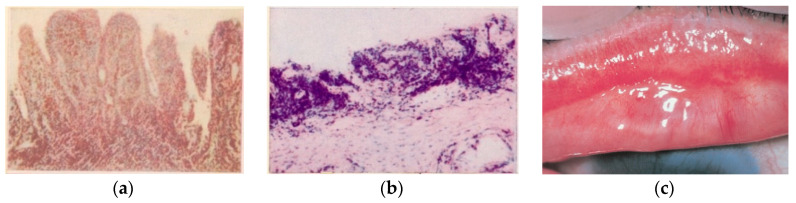
(**a**) Tarsal conjunctiva. Florid trachoma. (TF, according to WHO classification). Papillary infiltration, forming nodules. Van Gieson ferric hematoxylin. 75×. (**b**) Tarsal conjunctiva. Scarring trachoma. From TI (T. Inflammation Intense: fibroblastic proliferation of the chorion, still site of histiolymphocytic infiltration) to TS (T. Scarring): conjunctival scars with fibrous white bands (arrow). Absence of infiltrate and C*t* in the scar area. Giemsa 120× [16], (Courtesy Prof. Nicola Delle Noci). (**c**) Tarsal conjunctiva. (MacCallan T4); TS: Arlt’s line. (Reproduced with permission—reference number 230716-012078. From Weisenthal RW. *External Disease and Cornea.* Basic and Clinical Science Course, Section 8, American Academy of Ophthalmology, 2013–2014).

In the second half of the 1950s in Sicily island, another highly involved area, the experiences gathered in the Catania University Eye Clinic, directed by Prof. Giuseppe Favaloro, allowed Giuseppe Scuderi to publish a textbook and atlas of trachoma histopathology [17], clarifying from a clinical and anatomopathological point of view many aspects related to trachomatous infection (Figure 2a–c).

But to cut a long story short, here is a bird’s-eye summary of the major steps in Chlamydia research. 

Bedson in London, in a memorable review of viruses published in 1947, called this agent ‘*an obligate intracellular parasite with bacterial affinities’* that had a characteristic biological cliché and incorrectly named it *Bedsonia* [18]. The *Ct* cycle was first ‘illustrated’, and in a rudimentary manner, in 1938 at the Pasteur Institute in Algiers. At that time, *Ct*, the causative agent of ocular trachoma, was called *Rickettsia trachomatis* [19], as also quoted in GB Bietti [20], and in V. Cavara and GB. Bietti that presented the scientific report at the Italian Ophthalmological Society XXX national congress, held in Turin in 1952 [21]. The term “viruses” became apparent in 1965 with the advent of tissue culture and electron microscopy and the identification of bacterial ribosomal rRNA and cellular structures. However, the Chlamydiae were grouped under Rickettsiae until the genus *Chlamydia* was validated in 1966 [22]. Page and collaborators definitively described the biological cycle of *Chlamydia* with its distinguishing features, where the “Tang virus” has been quoted and named by the scientist Feifan Tang from Beijing, who manifested symptoms after inoculating himself with the laboratory specimen, isolated from infected monkeys, as published in *Acta Microbiologica Sinica* in 1956. 

## 2. Biology and Pathogenesis

*Chlamydia* is an obligate intracellular parasite as it is unable to synthesize ATP; it has long been considered a virus or rickettsia [21] or a protozoan, sensitive to treatment with penicillin [20]. Obtaining their growth on embryonic chicken eggs in 1957 and in cell culture in 1963 [23,24] gave the possibility to define their development cycle in two forms: Elementary Bodies (EBs) and Reticulated Bodies (RBs) and of taxonomic classification in the order Chlamydiales. EB represent the infectious, metabolically inactive stage, with a cell wall capable of resisting entities in the environment, like spores, penetrating the host’s epithelial cell by endocytosis, preventing the fusion of lysosomes with its own phagosome, thus allowing intracellular survival. The secretion of the glycogen mantle induces the transition to RB, a non-infectious vegetative stage, which multiplies by division as included bodies of 100–1000 RBs, obtaining energy by means of straw-like structures that cross the chlamys and penetrate the cytoplasm of the host epithelial cell. 

At the end of the incubation cycle, RBs re-differentiate into infecting EBs which are released by exocytosis or by rupture of the host cell [25]. The inflammatory response of the host that produces interferon-gamma leads to ‘alternate path’, a non-replicative phase defined as ‘persistent phase’, inducing depletion of tryptophan essential for the growth of the inclusion body. Re-differentiating into infecting EBs, the Chlamydiae can start a new developmental cycle. Trachoma and reactive arthritis are associated with the developmental cycle [26]. Infected epithelial cells release EBs which are phagocytosed by neutrophils via phagolysosomes and pro-inflammatory chemokines and cytokines [27], TNF-alpha and macrophage and granulocyte colony stimulating factor (GM-CSF). On-site studies conducted in Gambia, Nepal and Tanzania, respectively, confirmed the presence of elevated levels of TNF-alpha, important in maintaining inflammation and the subsequent development of scarring fibrosis [27], in particular of IL-6 and IL-15, and elevated levels of chemokines [28]. IL-17A appears to have a consistent role in promoting the pro-inflammatory response by triggering the inflammatory process that leads to tissue damage and scarring with fibrosis, up to Arlt’s line and entropion [29]. It still has to clarify the possibility that C*t* can trigger an autoimmune aggression process such as Yersinia or Shigella, suggesting also a possible onchogenetic role for ocular adnexal lymphoma [30]. 

A century after von Prowazek and Halberstädter’s discovery, little is still known about *Ct* and further studies are needed in order to discover its exact molecular mechanism of interaction with the host by subverting its innate immune defenses, facilitating chronic infection and intracellular survival.

In addition, a question is raised aimed at updating the other articles in this section of the Special Issue, along with epidemiology, transmission, duration of vaccination efficacy, vector and local microbiota. Because of differences in the microbiota between ocular and genital mucosal tissues, distinct serovars of *Ct* have adapted to these two tissue sites, particularly serovars A–C are topic for the ocular, while D–K for the genital tract. There is clear genetic and phenotypic evidence to explain this distinction. For example, the observation that the Ba sub-serovar is found in the genital tract correlates with its fully functional tryptophan synthetase, whereas all ocular strains have accumulated loss-of-function mutations in the synthetase gene. Is the emergence of Ba due to the repeated passages of B in the human genital tract, during which B re-acquires the function of its tryptophan synthetase gene? 

## 3. Laboratory

Chlamydiaceae are still a mystery: this “*ghost bacterium*” adapts to intracellular equilibrium, creating a chronic subclinical inflammation in the predisposed host, a sort of “*Inflammaging*” even in children, a long-lasting inflammation in the predisposed host, mainly HLA-B27-positive [31]. The ELISA test, although widely practiced, has low sensitivity, while nucleic acid amplification tests (NAAT, in particular RT-PCR) are considered the gold standard for specificity and sensitivity [32,33], while rapid immunochromatographic and immunofluorescence tests (IFA) have high specificity but even less sensitivity and, if negative, are still unreliable. However, the sampling method is critical: scraping is mandatory for chronic infections [34] to obtain sufficient quantities of infected cellular material. Four serotypes are responsible for human trachoma: A, B, Ba and C. Serotypes B,Ba, and D-K are responsible for inclusion conjunctivitis and *Ophtalmia neonatorum* [35]. 

**Figure 3:**
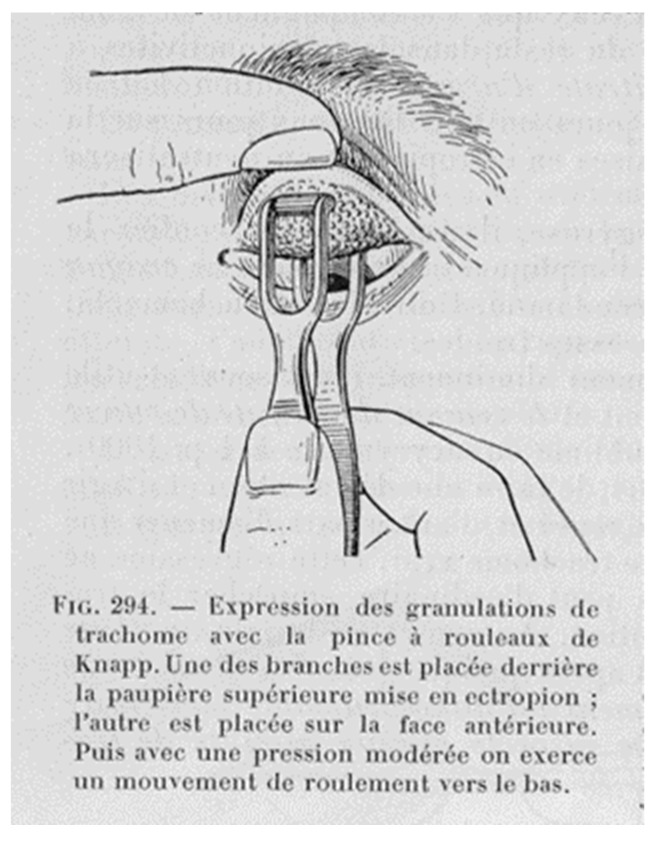
Knapp forceps for expression of the follicles of granular conjunctivitis. From Axenfeld [9], 1914. (Note: It is suggested to use gloves!).

In the florid forms, staining with May–Grumwald Giemsa on a slide, also with eyelid squeezing using Kuhnt or Knapp forceps (Figure 3), is indicative for inclusions identification and further differential diagnosis, especially in infections from multisite epithelial aggressors, such as gonococcus and *urogenital Mycoplasma* [36,37]. 

## 4. Contagion and Clinic, The SAFE-*S* Strategy

**Figure 4:**
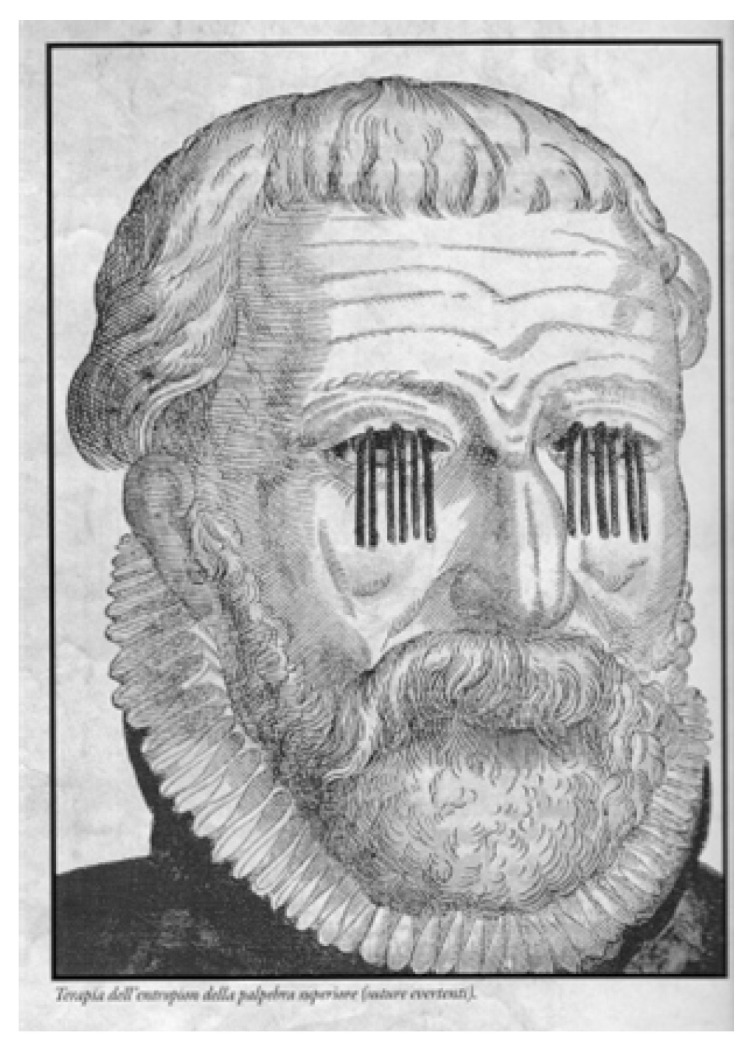
Everting sutures for entropion. George Bartisch: Ophthalmodouleia, 1583. Courtesy Prof. Nicola Delle Noci [40].

In the four clinical stages of trachoma (McCallan, 1908, cited by Frezzotti and Guerra [38]) from lymphoid hyperplasia to the florid highly contagious granulomatous stage 2—for which ancient treatments with massage, forceps, silver nitrate or copper sulfate brushing have found promoters and innovators over time—the evolution becomes complicated with the upper corneal “neovascular panniculus”. Leaving Herbert’s dimple in place of emptied limbal follicles, with large polynuclear macrophages and Moauro–Leber cells, the evolution of the disease follows the stages of xerophthalmia and tarsal scarring—Arlt’s line—inducing scar entropion, trichiasis and corneal ulcers with a likelihood of superinfection [4,7], causing blindness and needing surgery, but which is different from the past (Figure 4). However, the large burden of symptoms overlapping with other diseases or syndromes, and the priority identification as an exclusively ocular disease, delayed the identification of *Ct* by WHO as a sexually transmitted infection until 1976 [39]. 

Infection can be caused either directly or indirectly. In the indirect mechanism, flies, in particular, may be involved, which contribute to the spread of the disease by settling on the eyes of infected patients. Contact with eye or nasal secretions (including through cloths or clothing) of infected patients, on the other hand, constitutes a direct route of transmission of the disease.

The spread of the disease is inversely proportional to the socioeconomic development and sanitation conditions; in particular, risk factors can be identified in the “*lack of clean hot and running water, lack of personal cleanliness, crowding, promiscuity, superstitions, and abundance of flies*” [38]. Infection does not confer permanent immunity, so there is a likelihood of re-infection if the environmental causes of infection are not changed. An effective protocol of cyclic therapy protracted for four months to counteract the *Ct* biological cycle (EB-RB-EB) was developed by Gallenga PE et al., in 2018 (Table 1), achieving a complete microbiological and clinical resolution, leading to a favorable evolution in some cases of male (caused by chronic prostatitis) and female infertility, thus overcoming the “cultural era” to move to the “molecular era” [35].

Antimicrobial resistance is a major concern with the potential to cause millions of deaths, even though few studies recorded the clinical resistance of neglected tropical diseases (NTD), namely human African trypanosomiasis, leishmaniasis, onchocerciasis, schistosomiasis, soil-transmitted helminths, and trachoma [41]. Researches are in the pipeline to find which of the more than 2000 Antimicrobial peptides (AMPs) with broad-spectrum antibacterial, antiviral and antiparasitic activity, that have been shown to be effective against a variety of NTDs including trachoma, could be developed and used as alternatives [42,43]. Pep-1, LL-37, and melittin showed outstanding abilities in inhibiting the growth of *Ct.* The same applies to gene therapy: IRF5 and IL-10RA are related to macrophage–chlamydia interactions. Their roles seem vital in order to cure *Ct* infection in the scenario of the increasing antibiotic resistance, also to azytromycin. However, multiple challenges for the development of these therapies remained [43].

**Figure 5:**
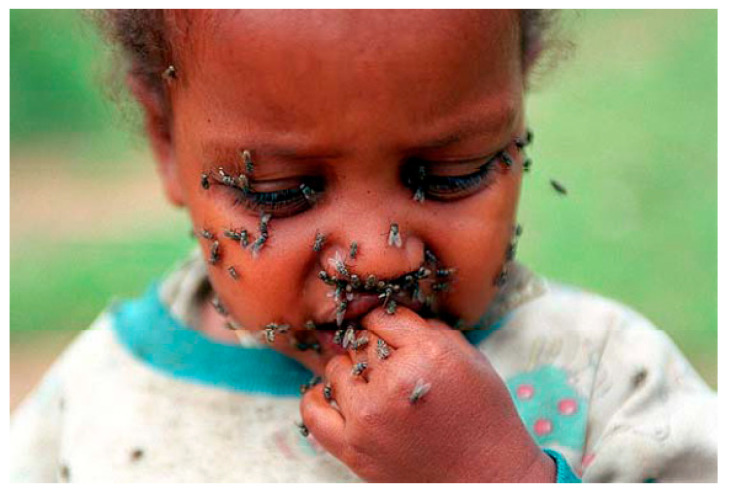
A Sri Lankan child with eyes infested with flies (*M. sorbens*). Courtesy Imperial College London. Institute of Global Health Innovation. The impact of Neglected Tropical Diseases on Universal eye health, 2016.

From the pictures of dirty and tearful children (Figure 5) in environments with poor sanitation in the Afro-Arabo-Indo-Asian basin, the contagion by flies is evident, although this does not account for the primitive forms of the disease in adults and those outside the tropical–equatorial area [36]. The WHO [44,45,46] and the Ligue contre le tTrachome (LCT) have rightly and successfully identified the SAFE strategy against trachoma (Surgery, Antibiotics, Facial cleanliness, Environmental improvement) as the blueprint for eliminating the disease by 2020; however, the evidence of the sexually transmitted disease still makes *Musca domestica*, *Musca sorbens* [47], *Scathophaga stercoraria* [48,49] or *Sarcophafaga carnaria*, so beloved by fishermen, the culprits. 

Having this scenario clear, we proposed to complete the acronym SAFE with –*S* (SAFE-*S*), understood as “strategy for controlling sexual well-being, i.e., sexual behavior [35].

## 5. Conclusions

**Figure 6:**
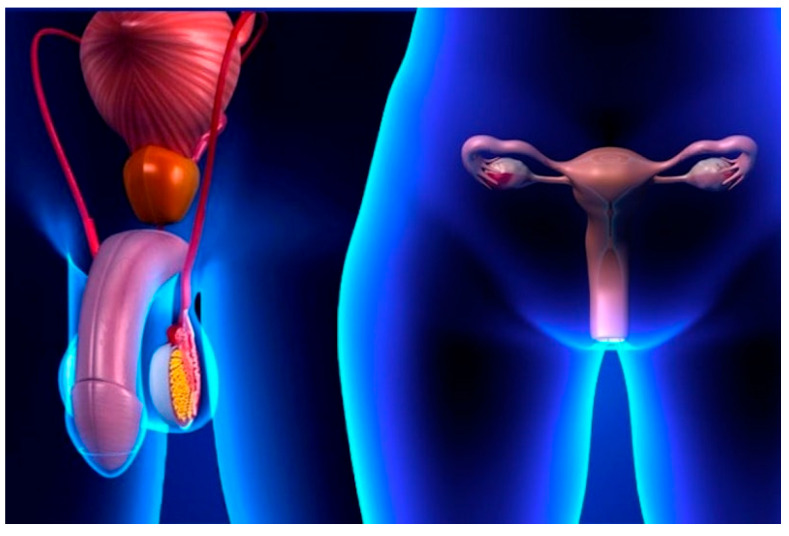
Unprotected sex is guilty. Improved control of sexual behavior is needed: SAFE-*S* strategy. Courtesy ihy-ihealthealthyou.com and digitaldictionary.it.

Although a large study has shown that the control of fly populations with insecticides reduces the prevalence of active trachoma disease [47], the WHO recently reported a major focus on sexual attitudes, which are changing in several areas of the world, leaving *Scatophaga stercoraria* and its fellow flies quite entirely blameless. But, extolling the role of *Ct* in apparently “sterile” chronic prostatitis, or conjunctivitis or, less frequently, oropharyngitis and proctitis, for which—as previously mentioned—it seems important to focus more on “sexual behaviour” [34,35] for the next decade [46], in order to attract the interest and attention of Obstetricians/Gynecologists, Urologists/Andrologists and Pediatricians. This means that sexually transmitted infections should be screened in asymptomatic patients with risky sexual behaviors or sexual contacts with people diagnosed with sexually transmitted infections. High school authorities should be mandated to improve students’ knowledge of sexually transmitted disease education and prophylaxis/protection (Figure 6).

The oculo-genital correlation and the need for adequate systemic coverage are confirmed by cases of chronic conjunctival disease resolved with ultrasound-guided infiltrative therapy of chronic prostatitis [50] or recurrent atypical chronic oropharyngeal inflammation resolved after chlamydial antibiotic therapy [51]. 

Therefore, we consider the occasion of this edition useful to relaunch an alert also for the Practitioners and the Social Physicians to holistically evaluate the patient, and in front of multisite infection “check for the presence of typical signs (Arlt’s line [5]; Neri’s white line [52]) or consequences: entropion and pannus, infertility [53], spontaneous abortion, pelvic inflammatory disease” [1,37], cervical cancer in association with HPV, and proctitis especially in homosexual males [33]. *Ct* also appears to be involved in oculo-orbital lymphoma [54], previously associated only with *Chlamydia psittaci*. New research would be desirable to confirm or exclude the hypothesis of *Chlamydia pneumoniae* as a chronic inflammatory stimulus for neovascular Age-related Macular Degeneration (nAMD) [55], inducing the inflammatory state [56]. Thus, it seems appropriate to draw the attention to *Chlamydia* for an evaluation of florid or chronic conjunctivitis, as well as for oropharyngeal diseases, such as *Neisseria gonorrhoeae* and other sexually transmitted diseases [57] which could also trigger, sometimes, reactive arthritis [58,59]. The PAHO-WHO plans to control the worldwide trachoma infection by 2020, between 2018 and 2019 showed reduction of 14,4 million people that lived in areas with high trachoma prevalence, but still leaving a total burden of 2,5 million cases of trachomatous trichiasis in 2019. This urgently requires an increase in medical and surgical strategy with decisive support for research and development of an effective and durable vaccine against chlamydial and mycoplasmal infections [60,61], improving the prevention by considering SAFE-*S* strategy, rather than insect powder. 

## Figures and Tables

**Table 1:** The first cycle (four months) for the annual planned protocol of anti-inflammatory (α-IT) and antibiotic (ATB) therapies, observing one month of pause before the complete cultural and molecular check [35].

a-IT and ATB	1st Week	2nd Week	3rd Week	4th Week
I cycle	CAF/HC ointment 3 times/8 h/day	AZT drop 3 times/8 h/day	N/D gel 3 times/8 h/day	1stcultural check
a-IT and ATB	5th week	6th week	7th week	8th week
II cycle	TC/SMT ointment 3 times/8 h/day	ERYsyrup 2 times/day	CAF/TC ointment 3 times/8 h/day	TC drop 3 times/8 h/day
a-IT and ATB	9th week	10th week	11th week	12th week
III cycle	AZT drop 3 times/8 h/day	CAF/HC ointment 3 times/8 h/day	TC drop 3 times/8 h/day	N/D gel 3 times/8 h/day
a-IT and ATB	13th week	14th week	15th week	16th week
Cultural and molecular check	No therapy	No therapy	No therapy	2nd control visit, cultural and molecular check

CAF/HC: Cloramphenicol/Hydrocortisone ointment 1% + 0.5%; AZT: Azithromycin drop 1.5%; N/D: Netilmicin/Dexamethasone gel 3 mg/mL + 1 mg/mL; TC/SMT: Tetracycline/ Sulphametiltiazole ointment 1% + 5%;ERY: Erythromycin syrup; CAF/TC: Chloramphenicol/Tetracycline ointment 1% + 0.5; TC: Tetracycline drop 1%.
